# A novel SlSTOP1–SlHAK5–cytosolic pH feedback loop drives dual adaptation to proton and aluminum toxicity in tomato

**DOI:** 10.1093/hr/uhaf241

**Published:** 2025-09-15

**Authors:** Huihui Zhu, Yuzhi Bai, Liqiong Jia, Lijie Jia, Feifei Liang, Chao Li, Zheng-An Yang, Wei Fan, Jianli Yang

**Affiliations:** Key Laboratory of Vegetable Biology of Yunnan Province, College of Landscape and Horticulture, Yunnan Agricultural University, Kunming 650201, China; Key Laboratory of Vegetable Biology of Yunnan Province, College of Landscape and Horticulture, Yunnan Agricultural University, Kunming 650201, China; Key Laboratory of Vegetable Biology of Yunnan Province, College of Landscape and Horticulture, Yunnan Agricultural University, Kunming 650201, China; College of Resource and Environment, Yunnan Agricultural University, Kunming 650201, China; Key Laboratory of Vegetable Biology of Yunnan Province, College of Landscape and Horticulture, Yunnan Agricultural University, Kunming 650201, China; Key Laboratory of Vegetable Biology of Yunnan Province, College of Landscape and Horticulture, Yunnan Agricultural University, Kunming 650201, China; Key Laboratory of Vegetable Biology of Yunnan Province, College of Landscape and Horticulture, Yunnan Agricultural University, Kunming 650201, China; Key Laboratory of Vegetable Biology of Yunnan Province, College of Landscape and Horticulture, Yunnan Agricultural University, Kunming 650201, China; College of Resource and Environment, Yunnan Agricultural University, Kunming 650201, China; Key Laboratory of Vegetable Biology of Yunnan Province, College of Landscape and Horticulture, Yunnan Agricultural University, Kunming 650201, China; College of Resource and Environment, Yunnan Agricultural University, Kunming 650201, China

## Abstract

Plants often experience aluminum (Al) toxicity in acidic soils, where the transcription factor SENSITIVE TO PROTON RHIZOTOXICITY1 (STOP1) plays a pivotal role in regulating transcriptional responses to Al stress. While posttranscriptional regulation of STOP1 under Al toxicity has been extensively studied, the mechanisms linking Al stress signals to STOP1 protein stability remain unclear. In this study, we employed multiscale pH imaging and noninvasive microtest (NMT) techniques to demonstrate that Al stress induces cytosolic acidification in the root apex of tomato (*Solanum lycopersicum*), which promotes the accumulation of SlSTOP1. This finding suggests that cytosolic acidification serves as a critical intermediate connecting Al stress to SlSTOP1 stabilization. Comparative transcriptomic analysis revealed that a significant subset of Al-responsive genes, including the known Al-resistance gene *SlHAK5*, are coregulated by both Al stress and low pH. Further functional characterization showed that *SlHAK5* not only contributes to Al resistance but also plays a key role in maintaining cytosolic pH homeostasis under Al stress. In *Slhak5* mutants, the expression of Al-induced genes was dysregulated, concomitant with attenuated cytosolic acidification. Correspondingly, SlSTOP1 accumulation was significantly reduced in *Slhak5* mutants compared to wild-type (AC) plants under Al stress, indicating that SlSTOP1-mediated *SlHAK5* expression feedback regulates cytosolic acidification. Additionally, *Slhak5* mutants exhibited heightened sensitivity to proton stress. Collectively, our findings uncover a novel regulatory circuit involving SlSTOP1 and SlHAK5, which modulates SlSTOP1 stability through cytosolic acidification, thereby enhancing plant adaptation to proton and Al toxicity.

## Introduction

Aluminum (Al), ranking as the third most abundant element in the Earth's crust after oxygen and silicon, constitutes a significant environmental stressor in acidic soils [1]. When soil pH drops below 5.5, the dissolution of insoluble Al-containing minerals releases phytotoxic Al^3+^ ions into the soil solution, which predominantly target root apices to suppress primary root elongation and overall plant development [2, 3]. This pH-dependent Al^3+^ mobilization poses a critical limitation to agricultural productivity, particularly given that acidic soils occupy approximately 40% of the world’s arable land [4].

To counteract Al^3+^ toxicity, plants have evolved two principal resistance strategies: external exclusion and internal detoxification [5]. Among these, the external exclusion mechanism, predominantly mediated by Al-activated root exudation of organic acids such as malate, citrate, and oxalate to chelate toxic Al^3+^ ions in the rhizosphere, has been extensively documented as the most pivotal Al resistance mechanism across diverse plant species [6, 7]. A landmark breakthrough in this field was the identification of the *Al-activated Malate Transporter 1* (*ALMT1*) gene from wheat through suppression subtractive cDNA library screening. This discovery not only unveiled the first *bona fide* Al resistance gene but also defined the ALMT protein family, a previously uncharacterized group of anion transporters [8]. Subsequently, map-based cloning approaches led to the isolation of citrate efflux transporters in barley (HvAACT1) and sorghum (SbMATE), both belonging to the multidrug and toxic compound extrusion (MATE) family [9, 10]. To date, extensive functional characterization of homologous genes across diverse plant species has further solidified the critical role of Al-activated organic acid (OA) secretion in plant Al resistance [7].

The C_2_H_2_-type zinc finger transcription factor STOP1 (SENSITIVE TO PROTON RHIZOTOXICITY1) was initially identified as a key regulator of proton (H^+^) and aluminum (Al) resistance mechanisms in Arabidopsis [11]. STOP1 controls the expression of multiple Al-tolerance genes, including *ALMT1*, *MATE*, and *ALS3* (*ALUMINUM SENSITIVE 3*), thereby contributing to both external and internal Al detoxification [12]. Interestingly, Al stress enhances STOP1 protein accumulation in roots posttranscriptionally, rather than at the transcriptional level [13]. STOP1 stability is tightly regulated by ubiquitin-mediated proteasomal degradation. The F-box protein RAE1 (REGULATION OF ATALMT1 EXPRESSION 1) targets STOP1 for degradation via the 26S proteasome pathway. RAE1 forms a complex with RHD6 (ROOT HAIR DEFECTIVE6) and GL2 (GLABRA2) to coregulate *ALMT1* expression [13, 14]. Additionally, SUMOylation of STOP1, mediated by Early in Short Days 4 (ESD4) and SIZ1, further modulates its activity [15, 16]. Recent studies have revealed that the MEKK1–MKK1/2-MPK4 kinase cascade enhances STOP1 phosphorylation and stability, promoting Al resistance [17]. Moreover, reactive oxygen species (ROS)-mediated oxidative modification has been implicated in STOP1 stability, adding another layer of regulation [18, 19].

Emerging evidence highlights the involvement of other factors in modulating STOP1 stability and nuclear localization. For instance, the phosphoinositide pathway, particularly PLC-mediated signaling, facilitates early STOP1 nuclear accumulation [20], suggesting that lipid-derived secondary messengers (e.g. IP₃, DAG) may prime STOP1 for transcriptional regulation. In align with Ca^2+^'s broader role in abiotic stress signaling, Ca^2+^ deprivation promotes STOP1 protein stability [21], implying that cytosolic Ca^2+^ fluxes suppress STOP1 under nonstress conditions. Under Pi deficiency, Fe availability (but not Pi deficiency alone) drives STOP1 nuclear accumulation [22]. Critically, low pH and Fe act synergistically, revealing a pH–Fe–STOP1 axis that integrates nutrient and stress signaling. In addition, NH₄^+^ uptake during Pi deficiency triggers rapid rhizosphere acidification, further amplifying STOP1 accumulation [23], linking nitrogen metabolism to Al resistance. Notably, it is well known that NO₃^−^ and K^+^ transport dynamically alter H^+^ homeostasis in root cells, potentially influencing STOP1 stability. We have previously demonstrated that the decreased citrate secretion in tomato *Slhak5* mutant was associated with reduced *SlFRDL1* expression, which contributes to the increased sensitivity of *Slhak5* to Al stress, but the molecular mechanism remains unknown [24]. Considering HAK5 (high-affinity K^+^ transporter 5) is a H^+^/K^+^ cotransporter, it is possible that cytosolic H^+^ homeostasis is implicated in SlHAK5-mediated *SlFRDL1* expression.

In the present study, we demonstrate that Al stress induces cytosolic acidification in tomato root apices, concomitant with reduced apoplastic acidification. This cytosolic acidification promotes the accumulation of SlSTOP1. Notably, both Al stress and low pH stress responses exhibit convergence, as evidenced by the overlapping expression patterns of Al resistance-related genes. Furthermore, we reveal that SlHAK5, a direct target of SlSTOP1, facilitates SlSTOP1 accumulation by regulating pH homeostasis, thereby establishing a SlSTOP1–SlHAK5–positive feedback loop that sustains SlSTOP1 levels. Our findings uncover a novel regulatory mechanism underlying plant adaptation to acidic soils, where H^+^ and Al stresses co-occur.

## Results

### Al^3+^ stress induces cytosolic acidification of tomato root apex

While STOP1 has been extensively characterized as a central regulator responsive to diverse abiotic stresses, emerging evidence suggests that cytosolic acidification may serve as an essential prerequisite for its activation [25]. Building upon our previous discovery that Al stress induces SlSTOP1 protein stabilization to transcriptionally activate at least 39 Al-responsive genes in tomato root apex [24], we postulated that Al-induced SlSTOP1 activation might be mechanistically associated with disruptions in H^+^ homeostasis. To investigate this hypothesis, we implemented a multiscale pH imaging approach to spatially resolve pH dynamics in both the rhizosphere and subcellular compartments of root apex cells.

**Figure 1:**
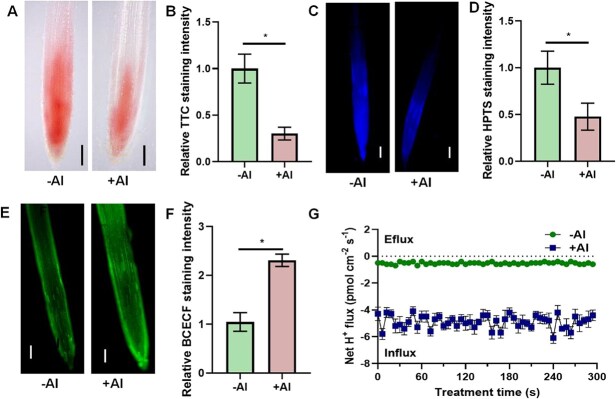
Al treatment induced cytosolic acidification of tomato root apex. (A, B) TTC staining of rhizosphere pH in AC root apex in response to Al treatment (A) and quantification of staining for A (B). (C, D) HPTS staining of apoplastic pH in AC root apex in response to Al treatment (A) and their quantification in C (D). (E, F) The BCECF fluorescent staining in AC root apex in response to Al treatment (A) and its statistical data (F). (G) H^+^ flux at the apical transition zone (ATZ) of the root apex with or without Al treatment. Seedlings with primary root length of 3 to 4 cm were subjected to 1/5 hoagland nutrient solution containing 0 (−Al) or 10 μM Al (+Al) for 6 h. After treatment, root apex was used for pH imaging and H^+^ flux measurement. Data are presented as the mean ± SD of six biological replicates per condition. Bar = 200 μm in (A), (C), and (E). Asterisks above the error bar indicate significant differences at *P* ≤ 0.05.

Using 2,3,5-triphenyltetrazolium chloride (TTC)-based colorimetric staining [26], we observed markedly reduced red formazan precipitation in the apical transition zone (ATZ) of Al-treated roots compared to controls (Fig. 1A and B), suggesting impaired H^+^ extrusion capacity under Al stress. Complementary 8-hydroxypyrene-1,3,6-trisulfonic acid trisodium salt (HPTS) ratiometric fluorescence imaging [27] revealed lower apoplastic acidification in Al-exposed roots (Fig. 1C and D). To determine whether these extracellular pH alterations were accompanied by intracellular changes, we employed the 2′,7′-bis-(2-carboxyethyl)-5-(and-6)-carboxyfluorescein, acetoxymethyl ester (BCECF) fluorescent probe [28] to monitor cytosolic pH in tomato root tips. Al treatment significantly increased fluorescence intensity, demonstrating cytoplasmic H^+^ accumulation (Fig. 1E and F). Furthermore, noninvasive microtest (NMT) technology revealed a net H^+^ influx at the distal transition zone of the root apex following Al treatment (Fig. 1G), consistent with the observed cytosolic acidification. Collectively, these findings demonstrate that Al stress induces significant cytosolic acidification in cells of tomato root apex.

### Low pH induces SlSTOP1 nuclear accumulation

**Figure 2:**
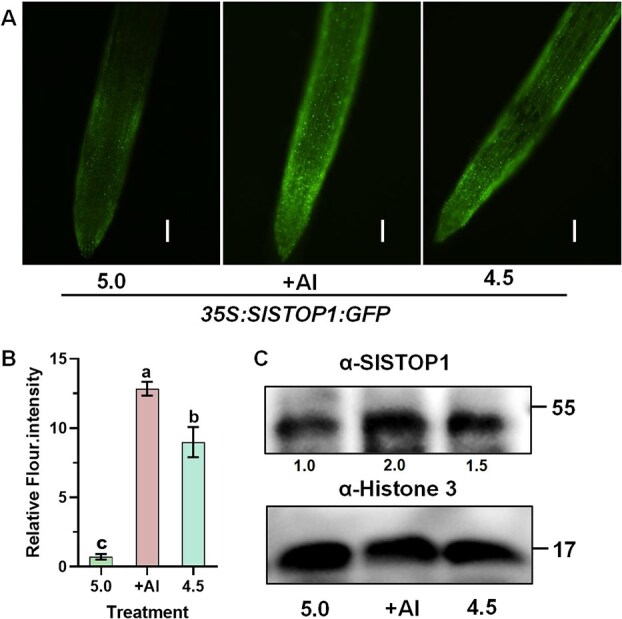
Low pH stress induced SlSTOP1 accumulation in tomato root apex. (A) SlSTOP1 accumulation by investigating GFP fluorescence intensity in *35S:SlSTOP1:GFP* transgenic reporter lines. (B) Quantification of GFP fluorescence from recombinant SlSTOP1–GFP protein in root tips. The *35S:SlSTOP1:GFP* transgenic reporter lines with primary root length of 3 to 4 cm were subjected to pH 5.0 (control), or 10 μM Al at pH 5.0 (+Al), or a lower pH of 4.5 for 6 h. GFP fluorescence was observed by fluorescence microscope. (C) Western blot analysis of SlSTOP1 accumulation. Tomato seedlings with primary root length of 3 to 4 cm were subjected to pH 5.0 (control), or 10 μM Al at pH 5.0 (+Al), or a lower pH of 4.5 for 6 h. Root tips (0–1 cm) were sampled for protein extraction. The same amount of total protein was then detected using antibodies raised against SlSTOP1 and Histone 3 (internal control). The different letters above the error bar indicate significant differences at *P* ≤ 0.05.

The finding that Al stress acidifies cytoplasm of root apex cells prompted us to examine whether acidification activates SlSTOP1 protein accumulation, whereby activating transcription of Al resistance genes. To test it, we first determined whether SlSTOP1 accumulation is pH dependent by taking advantage of transgenic tomato lines carrying *35S:SlSTOP1:GFP* construct. Compared to pH 5.0, a lower pH value of 4.5 (designated low pH stress hereafter) remarkably stimulated GFP signals, mirroring SlSTOP1 protein accumulation (Fig. 2A and B). For comparison, exposure of tomato roots to 10 μM Al was used as a positive control. Similar with low pH stress, Al treatment (pH 5.0) caused SlSTOP1 protein accumulation. We also determined SlSTOP1 protein accumulation under either Al treatment or low pH stress using antibodies raised against SlSTOP1 [29]. Obviously, SlSTOP1 protein was accumulated under Al treatment and low pH stress conditions, when compared to pH 5.0 (Fig. 2C). Furthermore, prolonged low pH treatment enhanced the nuclear accumulation of SlSTOP1 protein, demonstrating that acidic conditions promote SlSTOP1 translocation to the nucleus (Fig. S1).

### Low pH stress induces expression of Al-responsive genes

**Figure 3:**
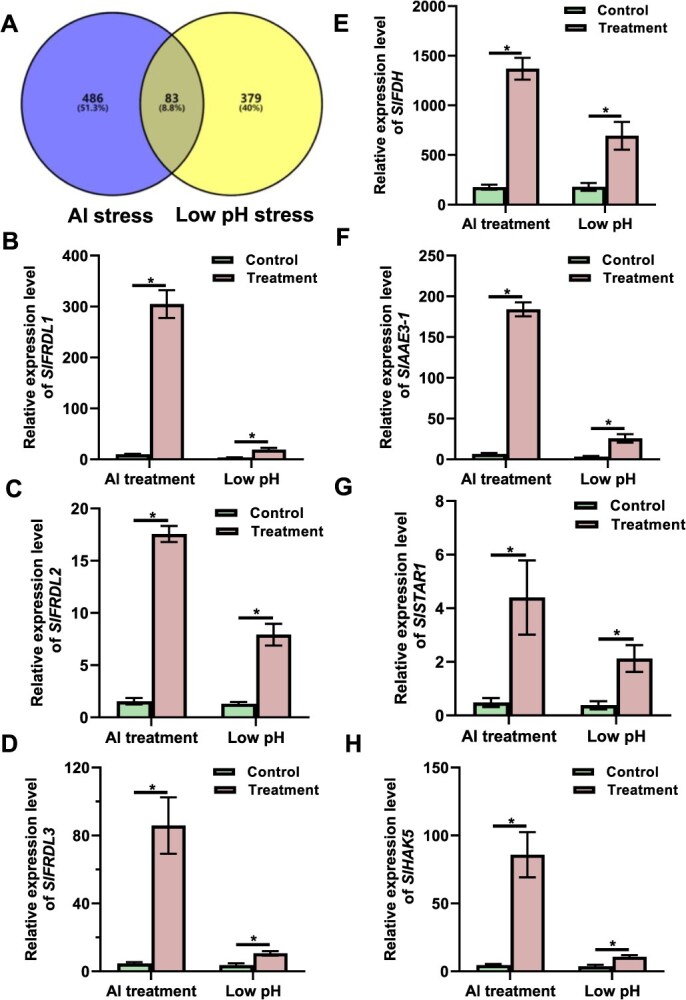
Response convergence between Al stress and low pH stress. (A) Venn diagram of overlapping genes between Al stress and low pH stress by comparative RNA-seq analysis. (B–H) qRT-PCR analysis of gene expression under Al stress or low pH stress. (B) *SlFRDL1*. (C) *SlFRDL2*. (D) *SlFRDL3*. (E) *SlFDH*. (F) *SlAAE3-1*. (G) *SlSTAR1*. (H) *SlHAK5*. Seedlings with primary root length of 3–4 cm were subjected to pH 5.0 (control), or 10 μM Al at pH 5.0 (+Al), or a lower pH of 4.5 for 6 h. Root apex (0–1 cm) were collected for RNA extraction. The expression level was calculated relative to *ACTIN*. Data are means ± SD (*n* = 3). Asterisks indicate significant differences at *P* ≤ 0.05.

Since cytosolic acidification serves as an intermediate process during Al stress, we hypothesized that Al-responsive genes might be transcriptionally activated under low pH stress conditions. To test this hypothesis, we conducted comparative RNA-seq analyses between low pH stress and Al stress. When tomato roots were exposed to 10 μM Al (+Al) for 6 h, we identified 569 upregulated genes (Table S1). In contrast, 462 genes were induced by low pH stress during the same duration (Table S2). A Venn diagram analysis revealed 83 overlapping genes between the two treatments, indicating a convergence in their stress responses (Fig. 3A, Table S3). KEGG pathway enrichment analysis revealed that these genes were mainly related to signaling pathways, ABC transporters, RNA processing, and various metabolic processes (Fig. S2). Notably, this overlapping set included canonical Al-responsive genes such as *SlFRDL1*, *SlFRDL2*, *SlFDH* (*formate dehydrogenase*), *SlAAE3-1* (*acyl-activating enzyme 3-1*), *SlSTAR1* (*sensitive to Al rhizotoxicity 1*), and *SlHAK5*. Subsequent qRT-PCR analysis confirmed that these genes were indeed upregulated under low pH conditions (Fig. 3B–H).

Our previous work demonstrated that SlSTOP1 directly regulates the expression of 39 Al-responsive genes [24]. Interestingly, among 83 overlapping genes, there are 19 genes that are SlSTOP1 targets, suggesting the convergence of two stresses is connected to SlSTOP1 (Table S3). To further explore the connection between Al-activated SlSTOP1 and cytosolic acidification, we investigated *SlHAK5*, one of the targets of SlSTOP1, promoter activity under low pH stress. Using *SlHAK5pro:GUS* transgenic lines in the AC background, we observed enhanced GUS expression in roots following Al treatment (Fig. S3A). Further analysis of *SlHAK5* expression under varying external pH levels revealed that its transcription was induced by low pH and intensified as pH decreased from 5.5 to 4.5 (Fig. S3B). Consistent with this, GUS activity was markedly stronger at pH 5.0 and 4.5 compared to pH 5.5 (Fig. S3C). Interestingly, when the pH of the Al treatment solution was adjusted to 5.5, GUS activity decreased significantly, suggesting that Al-induced *SlHAK5* expression is pH-dependent (Fig. S3D). These findings collectively demonstrate that cytosolic acidification plays a critical role in *SlHAK5* induction during Al stress.

### SlHAK5 mediates cytosolic pH homeostasis under Al stress

Building on our prior findings that SlHAK5 positively regulates Al resistance by modulating *SlFRDL1* expression and citrate secretion [24], we hypothesized that SlHAK5 may act as a molecular bridge connecting Al stress to cytosolic acidification. To investigate this, we systematically analyzed pH dynamics in both cytoplasmic and extracellular compartments of *Slhak5* mutants under Al exposure.

**Figure 4:**
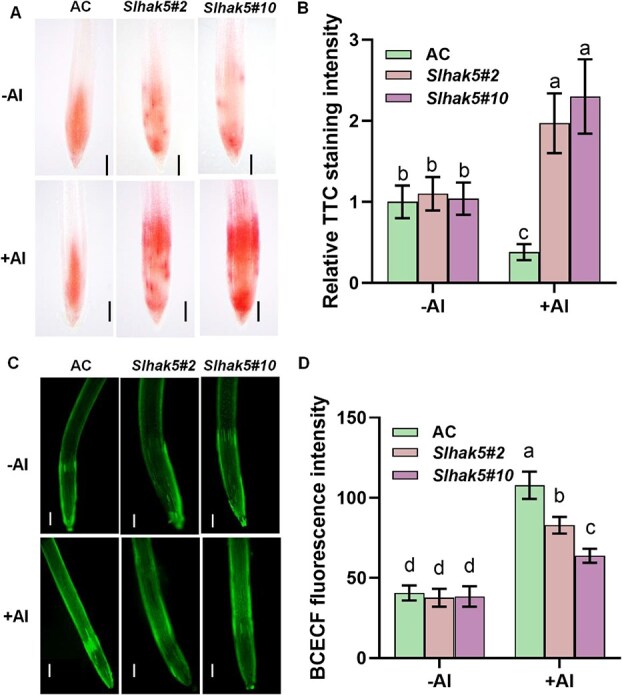
The apoplastic and cytosolic pH in roots of AC plants and two *Slhak5* mutants with or without Al treatment. (A) TTC staining of rhizosphere pH in roots of AC plants and two *Slhak5* mutant with and without Al treatment. (B) Quantification of TTC staining shown in (A). (C) The BCECF fluorescence in roots of AC plants and two *Slhak5* mutant without or with Al treatment. (D) Quantification of fluorescence intensity in (C). Seedlings with primary root length of 3–4 cm were subjected to 1/5 hoagland nutrient solution containing 0 (−Al) or 10 μM Al (+Al) for 6 h. After treatment, root apex was used for pH imaging. Data are presented as the mean ± SD of six biological replicates per condition in (D). Bar = 200 μm. The different letters above the error bar indicate significant differences at *P* ≤ 0.05.

Initial assessment of rhizosphere pH via TTC staining revealed no significant differences between wild-type (AC) and *Slhak5* mutants under −Al conditions. While Al treatment inhibited TTC staining in root apex of AC plants, it induced markedly stronger TTC staining in both mutants (Fig. 4A and B), indicating enhanced H^+^ efflux into the rhizosphere or reduced H^+^ influx in *Slhak5* mutants.

BCECF-based cytosolic pH monitoring showed comparable baseline fluorescence intensities in AC and *Slhak5* mutants under control conditions (pH 5.0, −Al; Fig. 4C and D). Upon Al treatment, all genotypes exhibited elevated fluorescence intensity (indicative of cytosolic acidification), but this response was significantly attenuated in *Slhak5* mutants (Fig. 4C). Quantitative analysis confirmed these differential responses (Fig. 4D). The observed phenotype aligns with a dual role for SlHAK5 in both limiting excessive H^+^ efflux and facilitating H^+^ influx, thereby facilitating cytosolic acidification. This mechanism complements its previously characterized function in regulating citrate secretion, i.e. mediating SlSTOP1 accumulation to induce expression of genes. Similarly, we measured apoplastic and cytosolic pH in both AC and *Slstop1* mutants under low pH conditions. Our results demonstrate that acidic treatment induced proton influx, leading to significant cytosolic acidification in AC root tips (Fig. S4A and B). In contrast, *Slstop1* mutants exhibited impaired cytosolic acidification (Fig. S4C and D), mirroring the phenotype previously observed in *Slhak5* mutants under Al stress.

### SlHAK5 modulates the expression of Al-responsive genes

The finding that SlHAK5 plays a crucial role in maintaining cytosolic pH homeostasis under Al stress suggests its important involvement in Al stress responses. To further investigate this, we performed comparative RNA sequencing of root tips (0–1 cm) between wild-type AC plants and *Slhak5* mutants. Under normal conditions (without Al treatment), only 65 differentially expressed genes (DEGs) were identified between AC and *Slhak5* mutants (Table S4). However, under Al stress, this number increased dramatically to 2946 DEGs (Table S5). To identify Al-induced genes potentially regulated by *SlHAK5*, we first compared 569 Al-induced genes in AC plants with the 65 DEGs identified in the ‘AC −Al vs *Slhak5* −Al’ comparison. This analysis revealed three overlapping genes: *SlHAK5* itself, a gene encoding suberization-associated anionic peroxidase 2, and one uncharacterized gene (Fig. S5A and B). Since hydrogen peroxide (H₂O₂) acts as a signaling molecule and has been reported to degrade STOP1 via oxidative modification [19], we investigated whether H₂O₂ contributes to *SlHAK5* induction under Al stress. To test this, we treated the AC and two *Slhak5* mutants with exogenous H₂O₂ alongside Al and analyzed *SlHAK5* expression. However, no significant difference was observed between treatments with or without H₂O₂ supplementation (Fig. S5C), indicating that *SlHAK5* expression is independent of H₂O₂-mediated signaling pathways. Furthermore, we identified 143 overlapping genes by comparing the 569 Al-induced DEGs in AC plants with the 2946 DEGs between AC and *Slhak5* mutants under Al treatment (Fig. S6). These genes could be classified into two distinct groups. One included 91 genes showing enhanced induction in *Slhak5* mutants, suggesting negative regulation by SlHAK5 (Fig. S6A). The other group included 52 genes exhibiting reduced expression in *Slhak5* mutants, indicating positive regulation by SlHAK5 (Fig. S6C). Gene ontology (GO) enrichment analysis revealed that the 91 SlHAK5-negatively regulated genes were significantly associated with the ‘lignin catabolic process’ pathway, while the 52 SlHAK5-positively regulated genes were predominantly enriched in the ‘ABC transporters’ pathway (Fig. S6B and D). These findings suggest that SlHAK5 plays a critical role in coordinating diverse metabolic processes in response to Al stress.

**Figure 5:**
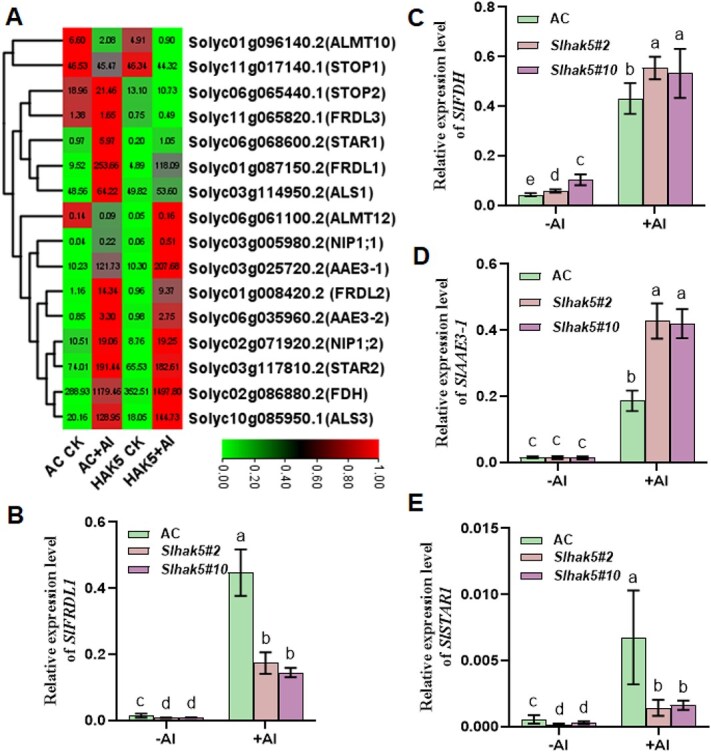
The influence of SlHAK5 mutation on the expression of canonical Al stress responsive genes. (A) Heatmap analysis of the canonical Al responsive genes between AC and *Slhak5* mutant with or without Al treatment. The values in heatmap were FPKM in RNA-seq. (B–E) qRT-PCR analysis the selected four genes for *SlFRDL1* (B), *SlFDH* (C), SlAAE3-1 (D), and SlSTAR1 (E). Seedlings with primary root length of 3–4 cm were subjected to 1/5 hoagland nutrient solution containing 0 (−Al) or 10 μM Al (+Al) for 6 h. Root apex (0–1 cm) were collected for RNA extraction. The expression level was calculated relative to *ACTIN*. Data are means ± SD (*n* = 3). The different letters above the error bar indicate significant differences at *P* ≤ 0.05.

To further elucidate the role of SlHAK5 in Al stress responses, we examined its influence on the expression of canonical Al-responsive genes. We selected 16 genes homologous to known Al-resistance genes for expression analysis (Fig. 5A). Notably, the expression of several key genes including *SlSTOP2*, *SlFRDL1*, *SlFRDL2*, *SlFRDL3*, *SlSTAR1*, and *SlALS1* was significantly suppressed in *Slhak5* mutants compared to wild-type AC plants under Al stress. In contrast, *SlALMT12*, *SlNIP1;1*, and *SlAAE3-1* exhibited upregulated expression in *Slhak5* mutants under the same conditions. To validate these findings, we performed qRT-PCR analysis on selected four genes involved in different processes of Al detoxification. SlFRDL1 that mediates citrate secretion is involved in chelating Al at rhizosphere [24]. SlSTAR1 is homologous to rice OsSTAR1 and buckwheat FeSTAR1 that modifies cell wall to reduce Al binding [30, 31]. Both SlFDH and SlAAE3-1 are two enzymes associated with acetylation degradation of cytoplasmic oxalate, which may be critical for regulating normal cellular function [32, 33]. The qRT-PCR results confirmed the RNA-seq data, demonstrating strong consistency between the two methods (Fig. 5B–E). Our previous work revealed that SlHAK5 is a direct target of SlSTOP1, which plays a crucial role in citrate secretion and Al tolerance in tomato [24]. The observed downregulation of SlFRDL1 in *Slhak5* mutants likely contributes to their reduced Al tolerance by impairing citrate efflux—a key mechanism for external Al detoxification. These findings further underscore the importance of SlHAK5 in regulating multiple Al-responsive pathways, mirroring its role in SlSTOP1 stability regulation.

### SlHAK5 feedback regulates SlSTOP1 protein stability

**Figure 6:**
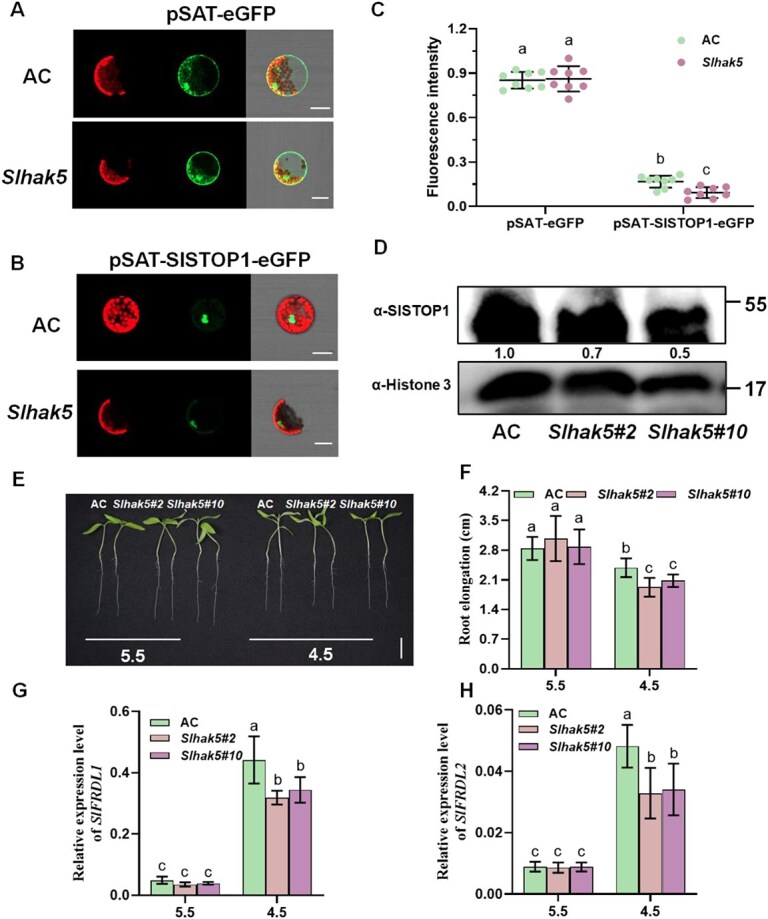
SlHAK5 positively regulates SlSTOP1 protein stability and H^+^ tolerance. (A) Fluorescence of eGFP proteins in protoplasts of AC plants and *Slhak5* mutant. (B) Fluorescence of SlSTOP1:GFP fusion proteins in protoplasts of AC plants and *Slhak5* mutant. (C) The statistical data of fluorescence intensity in (A, B). (D) SlSTOP1 accumulation in AC and two *Slhak5* mutant lines under Al treatment. AC and two *Slhak5* mutant lines with primary root length of 3 to 4 cm were subjected to Al treatment for 6 h. 1 cm root tips were samples for protein extraction. The same amount of crude protein was then detected using antibodies raised against SlSTOP1 and Histone 3. (E) The phenotype of representative seedlings AC plants and *Slhak5* mutant under low pH conditions. Bar = 1 cm. (F) The primary root elongation of AC and both *Slhak5* mutants in (E). (G, H) *SlFRDL1* and *SlFRDL2* expression in AC plants and *Slhak5* roots under low pH conditions. Tomato seedlings with primary root length of 3–4 cm were subjected to pH 5.5 or pH 4.5 for 2 days. Root length was measured before and after treatment. Data are means ± SD (*n* = 10). Root apex (0–1 cm) treated by low pH conditions for 6 h were collected for RNA extraction. The expression level was calculated relative to *ACTIN*. Data are means ± SD (*n* = 3). The different letters above the error bar indicate significant differences at *P* ≤ 0.05.

SlSTOP1 regulates *SlHAK5* expression in response to Al stress [24], which subsequently modulates the expression of Al-responsive genes (Fig. 5). This suggests a potential feedback loop in which SlHAK5 may regulate SlSTOP1 stability. To test this hypothesis, we conducted transient expression assays in leaf protoplasts derived from AC (wild type) and *Slhak5* mutants. When pSAT–eGFP was expressed in both AC and *Slhak5* protoplasts, no significant difference in GFP fluorescence intensity was observed (Fig. 6A and B). However, upon transient expression of the pSAT–SlSTOP1–eGFP fusion protein, GFP fluorescence localized exclusively to the nucleus, with markedly reduced intensity in *Slhak5* mutant protoplasts compared to AC (Fig. 6A–C). Further analysis revealed that SlSTOP1 protein accumulation in the root apex under Al stress was significantly lower in both *Slhak5* mutants than in AC (Fig. 6D). These findings demonstrate that SlHAK5 contributes to SlSTOP1 protein accumulation under Al stress, supporting the existence of a SlSTOP1–SlHAK5 positive feedback loop that maintains SlSTOP1 stability.

In addition to regulating Al resistance, SlSTOP1 has been shown to positively modulate H^+^ tolerance [24]. To further investigate the relationship between SlHAK5 function and SlSTOP1 protein stability, we assessed the role of SlHAK5 in H^+^ tolerance. We compared root elongation between wild-type AC plants and *Slhak5* mutants under low pH stress conditions. At pH 5.5, no significant difference in root elongation was observed between AC plants and the *Slhak5* mutants. However, under more acidic conditions, both *Slhak5* mutants exhibited significantly reduced root elongation compared to AC plants, demonstrating enhanced H^+^ sensitivity in the mutant lines (Fig. 6E and F). These results provide supporting evidence that SlHAK5 contributes to the stabilization of SlSTOP1 under both H^+^ and Al^3+^ stress conditions.

## Discussion

The transcription factor STOP1 plays a pivotal role in regulating the expression of aluminum (Al)-resistance genes in plants [7, 34]. While significant progress has been made in understanding the posttranscriptional regulation of STOP1, the mechanistic link between Al signal perception and STOP1 protein accumulation remains unclear. In this study, through the application of multiscale pH imaging techniques, we revealed that Al stress induces specific cytosolic acidification in the root apical region, concurrent with decreased acidification in both the apoplast and rhizosphere (Fig. 1). Importantly, our findings suggest that this cytosolic acidification likely facilitates SlSTOP1 accumulation, consequently activating downstream gene expression. This conclusion is substantiated by multiple lines of evidence. First, both Al stress and low pH conditions were found to promote SlSTOP1 accumulation (Fig. 2). Second, a considerable number of Al-responsive genes could also be induced by low pH stress (Fig. 3). Although the overlapping genes between these two treatments accounted for only 14.6% of total Al-responsive genes, key canonical Al-resistance genes, including direct targets of SlSTOP1 such as *SlHAK5*, were consistently upregulated by both stresses (Fig. S2). Further, it is hard to compare the signal intensity between low pH and Al stress. In fact, under our experimental condition, Al stress induced more abundant accumulation of SlSTOP1 protein than low pH-induced (Fig. 2). This may explain the expression induction of Al-responsive genes was less profound in low pH stress compared to Al stress (Fig. 3). Third, functional characterization demonstrated that *SlHAK5* positively regulates Al resistance by modulating the expression of other Al resistance genes, including *SlFRDL1* and *SlFDH* (Fig. 5). SlFRDL1, a member of the MATE-type citrate transporter family, has been shown to facilitate citrate efflux [32]. SlFDH, a mitochondrial-localized enzyme, catalyzes formate degradation while generating NADH, potentially contributing to ATP production during Al stress [33]. Notably, our results indicate that SlHAK5 regulates these Al resistance genes through its influence on cytosolic pH homeostasis and SlSTOP1 protein stability (Figs 4 and 6).

**Figure 7:**
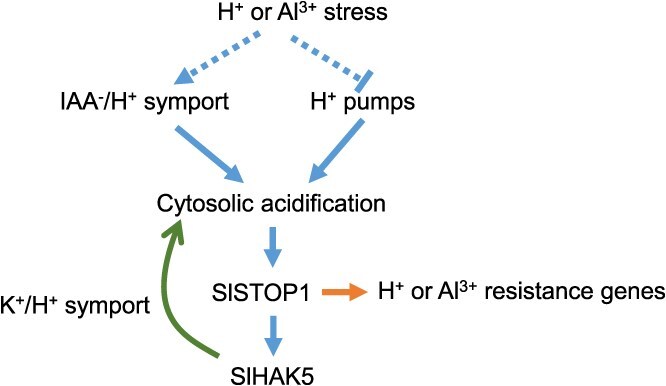
Schematic illustration of proton or Al stress induced cytosolic acidification in root apex of tomato. When root tips perceive H^+^ or Al^3+^ signals, they might drive concomitant H^+^ influx through IAA-H^+^ symporters, or experience dysregulation of plasma membrane H^+^-ATPase activity for inhibition of proton pumps, leading to pronounced cytosolic acidification (dashed lines represent putative processes). Al^3+^- or H^+^-induced cytosolic acidification in the root apex triggers SlSTOP1 protein accumulation to activate H^+^ or Al^3+^ resistance genes including *SlHAK5*. In turn, the upregulated *SlHAK5* expression promotes further cytosolic acidification through K^+^/H^+^ symport, thereby stabilizing SlSTOP1 itself.

Our previous work demonstrated that functional disruption of *SlHAK5* enhances Al sensitivity in mutants, concomitant with reduced citrate secretion [24]. Given the established role of cytosolic acidification in promoting SlSTOP1 accumulation, these findings strongly implicate SlHAK5 in modulating cytosolic pH dynamics. However, this raises a key mechanistic question: how does SlHAK5 regulate cytosolic pH homeostasis? As a plasma membrane-localized high-affinity K^+^ transporter, SlHAK5’s function in Al resistance appears independent of K^+^ deficiency [24]. Notably, experimental evidence from Arabidopsis and barley systems indicates that high-affinity K^+^ uptake occurs via H^+^-coupled transport, suggesting that AtHAK5 likely functions as a K^+^/H^+^ symporter in planta [35, 36]. Supporting this model, overexpression of *OsHAK5* in rice leads to measurable alkalization of the growth medium [37]. Collectively, these observations support a mechanism whereby SlHAK5 mediates cytosolic pH regulation through H^+^ cotransport during Al stress (Fig. 7).

Our findings align with and extend previous observations demonstrating that acidic conditions are essential for STOP1 activation and downstream gene expression. Several lines of evidence support this connection. First, experimental evidence showed that low pH alone can induce *ALMT1* expression independently of aluminum stress, suggesting H^+^ sensitivity in STOP1-mediated signaling [25]. Second, in acid soils, STOP1 is constitutively active and regulates *NRT1.1* expression, promoting rhizosphere alkalization as an adaptive response to low pH stress [38]. Third, under Pi-limiting conditions, STOP1-mediated *ALMT1* expression drives malate secretion from root apices, facilitating Fe^3+^ chelation and root system remodeling—a process that operates preferentially in acidic environments [22, 39, 40]. Notably, this response is further enhanced by NH₄^+^, a proton-releasing nitrogen source [23]. While these studies collectively establish extracellular acidity as a prerequisite for STOP1 activation across multiple stress responses, our work provides novel insight by demonstrating that cytosolic acidification may serve as the unifying trigger for STOP1 stabilization and subsequent signaling (Fig. 7).

While this study does not fully elucidate the mechanisms underlying Al-induced cytosolic acidification, we propose two plausible explanations based on existing evidence (Fig. 7). The first explanation is auxin-mediated H^+^ influx. We found that the root apical transition zone exhibits the most pronounced Al-induced cytosolic acidification (Fig. 4C), coinciding with the region where Al triggers auxin (IAA) accumulation [41, 42]. IAA uptake is mediated by AUX/LAX transporters, which function as IAA-H^+^ symporters [43]. Thus, Al-induced IAA accumulation may drive concomitant H^+^ influx, consistent with our observation of enhanced H^+^ uptake in the transition zone (Fig. 1). The second explanation could be dysregulation of H^+^ pumps. Plasma membrane (PM) H^+^–ATPases extrude protons to establish electrochemical gradients, but Al may perturb their activity. For instance, Al induces hyperpolarization (increased H^+^–ATPase activity) in Al-resistant wheat but depolarization (inhibition of H^+^–ATPase activity) in Al-sensitive genotypes [44]. Similar variability occurs in soybean and faba bean [45–47]. Besides, tonoplast H^+^-PPases and H^+^-ATPases could also contribute to cytosolic pH shifts. A recent study on apple (*Malus domestica*) indicated that MdSTOP1 interacts with MdNAC2 to regulate *MdNXH2* (*SODIUM HYDROGEN EXCHANGER 2*), which facilitates H^+^ efflux from the vacuole to the cytoplasm (cytosolic acidification) [48]. Given the interplay between auxin transport and PM H^+^-ATPase activity (e.g. auxin-triggered ATPase activation), we propose that both mechanisms synergize to induce cytosolic acidification under Al stress. Future studies should dissect their relative contributions.

In summary, our findings demonstrate that Al-induced cytosolic acidification in the root apex, particularly within the transition zone, triggers SlSTOP1 protein accumulation, leading to transcriptional activation of key genes involved in Al^3+^ and H^+^ tolerance. Crucially, we reveal a self-reinforcing regulatory mechanism whereby SlSTOP1 upregulates *SlHAK5* expression, which in turn promotes further cytosolic acidification to stabilize SlSTOP1. This positive feedback loop represents an adaptive strategy that simultaneously enhances tomato's tolerance to both Al^3+^ toxicity and H^+^ stress (Fig. 7).

## Materials and methods

### Plant materials and treatment conditions

The tomato (*Solanum lycopersicum*) cultivar Ailsa Craig (AC), CRISPR/Cas9-based *Slhak5* mutants and *35S:SlSTOP1:GFP* transgenic reporter lines used in this study has been described in our previous study [24]. To construct the *SlHAK5_Pro_:GUS* transgenic plants, a 2-kb promoter of *SlHAK5* was cloned into pCAMBIA1300-NosP:HPTGUS Plus vector and transformed into AC plants via hygromycin selection to obtain homozygous transgenic plants. Seeds were surface sterilized by 10% NaClO (v/v) for 15 min. After washed with sterilized water thoroughly, the seeds were immersed in sterilized water overnight and then transferred to petri dishes containing 1/5 Hoagland nutrient solution (pH 5.5) and 0.8% agar with a 16-h day and 8-h night photoperiod. Temperature was 24°C at daytime and 22°C at night. Seedlings with primary root in 3 to 4 cm length were selected for treatments. All treatments were conducted in hydroponics as previous reported [19]. In brief, for Al resistance assay, seedlings were subjected to 1/5 Hoagland nutrient solution (pH 5.0) with (NH_4_)H_2_PO_4_ concentration decreased to 10 μM either in the absence (−Al) or presence (+Al) of 10 μM Al for different treatment duration. For different pH stresses, seedlings were subjected to nutrient solution adjusted to pH 5.5, 5.0, or 4.5.

### Quantitative real-time PCR and RNA-seq analysis

After treatment, root apices (0–1 cm) were collected for RNA extraction. Total RNA was extracted by RNAprep Pure Plant Kit and then transcribed into cDNA using PrimeScript RT Master Mix. SYBR Green serving as the detection dye was used to performed qPCR assay by a LightCycler 480 device. *ACTIN* was used as an internal control to normalize expression levels for each gene. The primer information is shown in Table S6. RNAseq was performed on the Illumina HiSeq platform with three biological replicates for each treatment, as described in a previous study [49].

### pH imaging and measurement of net H^+^ flux in root tips

The pH indicator HPTS was used for apoplast pH staining [50]. The roots of AC and two *Slhak5* mutants without or with Al treatment were incubated in 1 mM HPTS (pH 5) for 30 min and then rinsed with water three times before imaging. Images were collected at 510 nm with a plate reader (excitation wavelength = 460 nm). To assay intracellular pH in the root tips, the intracellular pH indicator BCECF-AM (Med Chem Express, Shanghai, China) was used. Root tips after Al treatments were incubated with 10 μM BCECF-AM for 1 h in darkness at 22°C and then rinsed with water before imaging. Images were taken at 535 to 550 nm emission after excitation at 488 nm by confocal microscope. The TTC method was used to qualitatively assess rhizosphere H^+^ according to [48].

The net H^+^ fluxes were measured by noninvasive microtest technology (NMT, Xuyue Beijing Science and Technology Company). The NMT system is NMT150S and the software is imFluxesV2.0 (Younger USA LLC, Amherst, MA 01002, USA). Briefly, about 3 to 4 cm primary roots of AC were treated without or with Al treatment for 6 h. Then, the H^+^ fluxes were measured using an H^+^-selective microelectrode with six biological replicates for each treatment.

### Transient expression analysis in tomato leaf protoplast and GFP fluorescence observation

To investigate the SlSTOP1 protein accumulation in *Slhak5* mutants, we extracted protoplasts from leaves of AC and *Slhak5* mutants and then transiently expressed SlSTOP1 in these protoplasts. The ORFs of SlSTOP1 without ending codon were amplified and then cloned into pSAT6–eGFP vector. The protoplast transformation was performed according to the polyethylene glycol (PEG) method and then visualized the GFP fluorescence via fluorescence microscopy. Similarly, the GFP fluorescence was also observed in the root tips of *35S:SlSTOP1:GFP* transgenic plants under low pH condition. The fluorescence intensity was subsequently quantified by using Image J software.

### Western blot

To investigate SlSTOP1 protein accumulation in AC and *Slhak5* mutants, total protein was extracted from the root tips of AC and *Slhak5* mutants. The polyclonal antibody raised against STOP1 and anti-rabbit IgG HRP-conjugated antibody as the primary antibody and secondary antibody, respectively, were purchased from Abclonal (Wuhan, China). An anti-Histone 3 antibody (Sigma) was used to normalize the loading quantity of the proteins.

### Statistical analysis

The data are presented as means ± standard deviation (SD) derived from four to six biological replicates across various assays. Statistical analyses were conducted using one-way or two-way ANOVA, with *P* ≤ 0.05 indicating significant differences between groups.

## Supplementary Material

Web_Material_uhaf241

## Data Availability

RNA-Seq data is available as accession number PRJNA1052400 for AC and *Slhak5* mutants in response to Al stress treatment, and PRJNA1255724 for low pH stress of AC plants in the NCBI SRA database (https://www.ncbi.nlm.nih.gov).

## References

[1] Delhaize E, Ryan PR. Aluminum toxicity and tolerance in plants. Plant Physiol. 1995;107:315–21 12228360 10.1104/pp.107.2.315 PMC157131

[2] Ma JF . Syndrome of aluminum toxicity and diversity of aluminum resistance in higher plants. Int Rev Cytol. 2007;264:225–52 17964924 10.1016/S0074-7696(07)64005-4

[3] Zheng SJ, Yang JL. Target sites of aluminum phytotoxicity. Biol Plant. 2005;49:321–31

[4] Kochian LV, Pineros MA, Liu J. et al. Plant adaptation to acid soils: the molecular basis for crop aluminum resistance. Annu Rev Plant Biol. 2015;66:571–98 25621514 10.1146/annurev-arplant-043014-114822

[5] Ma JF, Ryan PR, Delhaize E. Aluminium tolerance in plants and the complexing role of organic acids. Trend Plant Sci. 2001;6:273–8 10.1016/s1360-1385(01)01961-6 11378470

[6] Kochian LV, Hoekenga OA, Pineros MA. How do crop plants tolerate acid soils? Mechanisms of aluminum tolerance and phosphorous efficiency. Annu Rev Plant Biol. 2004;55:459–93 15377228 10.1146/annurev.arplant.55.031903.141655

[7] Yang JL, Fan W, Zheng SJ. Mechanisms and regulation of aluminum-induced secretion of organic acid anions from plant roots. J Zhejiang Univ-Sci B. 2019;20:513–27 31090277 10.1631/jzus.B1900188 PMC6568218

[8] Sasaki T, Yamamoto Y, Ezaki B. et al. A wheat gene encoding an aluminum-activated malate transporter. Plant J. 2004;37:645–53 14871306 10.1111/j.1365-313x.2003.01991.x

[9] Furukawa J, Yamaji N, Wang H. et al. An aluminum-activated citrate transporter in barley. Plant Cell Physiol. 2007;48:1081–91 17634181 10.1093/pcp/pcm091

[10] Magalhaes JV, Liu J, Guimaraes CT. et al. A gene in the multidrug and toxic compound extrusion (MATE) family confers aluminum tolerance in sorghum. Nat Genet. 2007;39:1156–61 17721535 10.1038/ng2074

[11] Iuchi S, Koyama H, Iuchi A. et al. Zinc finger protein STOP1 is critical for proton tolerance in Arabidopsis and coregulates a key gene in aluminum tolerance. Proc Natl Acad Sci USA. 2007;104:9900–5 17535918 10.1073/pnas.0700117104 PMC1887543

[12] Sawaki Y, Iuchi S, Kobayashi Y. et al. STOP1 regulates multiple genes that protect Arabidopsis from proton and aluminum toxicities. Plant Physiol. 2009;150:281–94 19321711 10.1104/pp.108.134700 PMC2675709

[13] Zhang Y, Zhang J, Guo JL. et al. F-box protein RAE1 regulates the stability of the aluminum-resistance transcription factor STOP1 in Arabidopsis. Proc Natl Acad Sci USA. 2019b;116:319–27 30559192 10.1073/pnas.1814426116 PMC6320511

[14] Cao HR, Zhang M, Zhu X. et al. The RAE1-STOP1-GL2-RHD6 module regulates the ALMT1-dependent aluminum resistance in Arabidopsis. Nat Commun. 2024;15:6294 39060273 10.1038/s41467-024-50784-1 PMC11282296

[15] Fang Q, Zhang J, Zhang Y. et al. Regulation of aluminum resistance in Arabidopsis involves the SUMOylation of the zinc finger transcription factor STOP1. Plant Cell. 2020;32:3921–38 33087527 10.1105/tpc.20.00687 PMC7721324

[16] Xu JM, Zhu JY, Liu JJ. et al. SIZ1 negatively regulates aluminum resistance by mediating the STOP1–ALMT1 pathway in Arabidopsis. J Integr Plant Biol. 2021;63:1147–60 33710720 10.1111/jipb.13091

[17] Zhou F, Singh S, Zhang J. et al. The MEKK1-MKK1/2-MPK4 cascade phosphorylates and stabilizes STOP1 to confer aluminum resistance in Arabidopsis. Mol Plant. 2022;16:337–53 36419357 10.1016/j.molp.2022.11.010

[18] Ding ZJ, Xu C, Yan JY. et al. The LRR receptor-like kinase ALR1 is a plant aluminum ion sensor. Cell Res. 2024;34:281–94 38200278 10.1038/s41422-023-00915-y PMC10978910

[19] Wei X, Zhu Y, Xie W. et al. H_2_O_2_ negatively regulates aluminum resistance via oxidation and degradation of the transcription factor STOP1. Plant Cell. 2024;36:688–708 37936326 10.1093/plcell/koad281 PMC10896299

[20] Tokizawa M, Enomoto T, Ito H. et al. High affinity promoter binding of STOP1 is essential for early expression of novel aluminum-induced resistance genes GDH1 and GDH2 in Arabidopsis. J Exp Bot. 2021;72:2769–89 33481007 10.1093/jxb/erab031

[21] Tian WH, Cai WY, Zhu CQ. et al. STOP1 regulates CCX1-mediated Ca^2+^ homeostasis for plant adaptation to Ca^2+^ deprivation. J Integr Plant Biol. 2024;66:2126–39 39092784 10.1111/jipb.13754

[22] Godon C, Mercier C, Wang X. et al. Under phosphate starvation conditions, Fe and Al trigger accumulation of the transcription factor STOP1 in the nucleus of Arabidopsis root cells. Plant J. 2019;99:937–49 31034704 10.1111/tpj.14374 PMC6852189

[23] Tian WH, Ye JY, Cui MQ. et al. A transcription factor STOP1-centered pathway coordinates ammonium and phosphate acquisition in Arabidopsis. Mol Plant. 2021;14:1554–68 34216828 10.1016/j.molp.2021.06.024

[24] Zhu H, Chen W, Za Y. et al. SlSTOP1-regulated SlHAK5 expression confers Al tolerance in tomato by facilitating citrate secretion from roots. Hortic Res. 2024;11:uhae282 39545040 10.1093/hr/uhae282 PMC11561044

[25] Le Poder L, Mercier C, Fevrier L. et al. Uncoupling aluminum toxicity from aluminum signals in the STOP1 pathway. Front Plant Sci. 2022;13:785791 35592558 10.3389/fpls.2022.785791 PMC9111536

[26] Gong XQ, Shi ST, Dou FF. et al. Exogenous melatonin alleviates alkaline stress in *Malus hupehensis* Rehd. by regulating the biosynthesis of polyamines. Molecules. 2017;22:1542 28902159 10.3390/molecules22091542 PMC6151414

[27] Zhang F, Yan XY, Han XB. et al. A defective vacuolar proton pump enhances aluminum tolerance by reducing vacuole sequestration of organic acids. Plant Physiol. 2019a;181:743–61 31350362 10.1104/pp.19.00626 PMC6776860

[28] Ozkan P, Mutharasan R. A rapid method for measuring intracellular pH using BCECF-AM. BBA-Gen Subjects. 2002;1572:143–8 10.1016/s0304-4165(02)00303-3 12204343

[29] Zhang L, Dong D, Wang J. et al. A zinc finger protein SlSZP1 protects SlSTOP1 from SlRAE1-mediated degradation to modulate aluminum resistance. New Phytol. 2022;236:165–81 35739643 10.1111/nph.18336

[30] Huang CF, Yamaji N, Mitani N. et al. A bacterial-type ABC transporter is involved in aluminum tolerance in rice. Plant Cell. 2009;21:655–67 19244140 10.1105/tpc.108.064543 PMC2660611

[31] Xu JM, Lou HQ, Jin JF. et al. A half-type ABC transporter FeSTAR1 regulates Al resistance possibly via UDP-glucose-based hemicellulose metabolism and Al binding. Plant Soil. 2018;432:303–14

[32] Jin JF, Zhu HH, He QY. et al. The tomato transcription factor SlNAC063 is required for aluminum tolerance by regulating *SlAAE3-1* expression. Front Plant Sci. 2022;13:826954 35371150 10.3389/fpls.2022.826954 PMC8965521

[33] He QY, Jin JF, Li PF. et al. Involvement of SlSTOP1 regulated *SlFDH* expression in aluminum tolerance by reducing NAD^+^ to NADH in the tomato root apex. Plant J. 2023;113:387–401 36471650 10.1111/tpj.16054

[34] Huang CF, Ma Y. Aluminum resistance in plants: a critical review focusing on STOP1. Plant Commun. 2024;6:101200 39628052 10.1016/j.xplc.2024.101200 PMC11897453

[35] Maathuis FJM, Sanders D. Mechanism of high-affinity potassium uptake in roots of Arabidopsis thaliana. Proc Natl Acad Sci USA. 1994;91:9272–6 7937754 10.1073/pnas.91.20.9272 PMC44794

[36] Walker DJ, Leigh RA, Miller AJ. Potassium homeostasis in vacuolate plant cells. Proc Natl Acad Sci USA. 1996;93:10510–4 11607707 10.1073/pnas.93.19.10510 PMC38416

[37] Yang TY, Feng HM, Zhang S. et al. The potassium transporter OsHAK5 alters rice architecture via ATP-dependent transmembrane auxin fluxes. Plant Commun. 2020;1:100052 33367257 10.1016/j.xplc.2020.100052 PMC7747981

[38] Ye JY, Tian WH, Zhou M. et al. STOP1 activates NRT1.1-mediated nitrate uptake to create a favorable rhizospheric pH for plant adaptation to acidity. Plant Cell. 2021;33:3658–74 34524462 10.1093/plcell/koab226 PMC8643680

[39] Balzergue C, Dartevelle T, Godon C. et al. Desnos T (2017) low phosphate activates STOP1–ALMT1 to rapidly inhibit root cell elongation. Nat Commun. 2017;8:15300 28504266 10.1038/ncomms15300 PMC5440667

[40] Xu JM, Wang ZQ, Wang JY. et al. Low phosphate represses histone deacetylase complex1 to regulate root system architecture remodeling in Arabidopsis. New Phytol. 2020;225:1732–45 31608986 10.1111/nph.16264

[41] Kollmeier M, Felle HH, Horst WJ. Genotypical differences in aluminum resistance of maize are expressed in the distal part of the transition zone. Is reduced basipetal auxin flow involved in inhibition of root elongation by aluminum? Plant Physiol. 2000;122:945–56 10712559 10.1104/pp.122.3.945 PMC58931

[42] Yang ZB, Geng XY, He CM. et al. TAA1-regulated local auxin biosynthesis in the root-apex transition zone mediates the aluminum-induced inhibition of root growth in Arabidopsis. Plant Cell. 2014;26:2889–904 25052716 10.1105/tpc.114.127993 PMC4145121

[43] Spalding EP . Diverting the downhill flow of auxin to steer growth during tropisms. Am J Bot. 2013;100:203–14 23284058 10.3732/ajb.1200420

[44] Ahn SJ, Rengel Z, Matsumoto H. Aluminum-induced plasma membrane surface potential and H^+^-ATPase activity in near-isogenic wheat lines differing in tolerance to aluminum. New Phytol. 2004;162:71–9

[45] Shen H, He LF, Sasaki T. et al. Citrate secretion coupled with the modulation of soybean root tip under aluminum stress.: up-regulation of transcription, translation, and threonine-oriented phosphorylation of plasma membrane H^+^-ATPase. Plant Physiol. 2005;138:287–96 15834009 10.1104/pp.104.058065 PMC1104183

[46] Kim YS, Park W, Nian H. et al. Aluminum tolerance associated with enhancement of plasma membrane H+-ATPase in the root apex of soybean. Soil Sci Plant Nutr. 2010;56:140–9

[47] Chen Q, Guo CL, Wang P. et al. Up-regulation and interaction of the plasma membrane H^+^-ATPase and the 14-3-3 protein are involved in the regulation of citrate exudation from the broad bean (*Vicia faba* L.) under Al stress. Plant Physiol Biochem. 2013;70:504–11 23860230 10.1016/j.plaphy.2013.06.015

[48] Wang C, Bian C, Li J. et al. Melatonin promotes Al^3+^ compartmentalization via H^+^ transport and ion gradients in *Malus hupehensis*. Plant Physiol. 2023;193:821–39 37311207 10.1093/plphys/kiad339

[49] Zhu HH, Wang JY, Jiang D. et al. The miR157-SPL-CNR module acts upstream of bHLH101 to negatively regulate iron deficiency responses in tomato. J Integr Plant Biol. 2022;64:1059–75 35297168 10.1111/jipb.13251

[50] Barbez E, Dünser K, Gaidora A. et al. Auxin steers root cell expansion via apoplastic pH regulation in *Arabidopsis thaliana*. Proc Natl Acad Sci USA. 2017;114:e4884–93 28559333 10.1073/pnas.1613499114 PMC5474774

